# Simvastatin ameliorates established pulmonary hypertension through a heme oxygenase-1 dependent pathway in rats

**DOI:** 10.1186/1465-9921-10-32

**Published:** 2009-05-02

**Authors:** Hsao-Hsun Hsu, Wen-Je Ko, Jo-Yu Hsu, Jin-Shing Chen, Yung-Chie Lee, I-Rue Lai, Chau-Fong Chen

**Affiliations:** 1 Department of Surgery, National Taiwan University Hospital and National Taiwan University College of Medicine, Taipei, Taiwan, ROC; 2 Graduate Institute of Physiology, College of Medicine, National Taiwan University, Taipei, Taiwan, ROC; 3 Department of Traumatology, National Taiwan University Hospital and National Taiwan University College of Medicine,Taipei, Taiwan, ROC

## Abstract

**Background:**

Simvastatin has been shown to ameliorate pulmonary hypertension by several mechanisms in experimental animal models. In this study, we hypothesized that the major benefits of simvastatin in pulmonary hypertension occur via the heme oxygenase-1 pathway.

**Methods:**

Simvastatin (10 mg/kgw/day) was tested in two rat models of pulmonary hypertension (PH): monocrotaline administration and chronic hypoxia. The hemodynamic changes, right heart hypertrophy, HO-1 protein expression, and heme oxygenase (HO) activity in lungs were measured in both models with and without simvastatin treatment. Tin-protoporphyrin (SnPP, 20 μmol/kg w/day), a potent inhibitor of HO activity, was used to confirm the role of HO-1.

**Results:**

Simvastatin significantly ameliorated pulmonary arterial hypertension from 38.0 ± 2.2 mm Hg to 22.1 ± 1.9 mm Hg in monocrotaline-induced PH (MCT-PH) and from 33.3 ± 0.8 mm Hg to 17.5 ± 2.9 mm Hg in chronic hypoxia-induced PH (CH-PH) rats. The severity of right ventricular hypertrophy was significantly reduced by simvastatin in MCT-PH and CH-PH rats. Co-administration with SnPP abolished the benefits of simvastatin. Simvastatin significantly increased HO-1 protein expression and HO activity in the lungs of rats with PH; however co-administration of SnPP reduced HO-1 activity only. These observations indicate that the simvastatin-induced amelioration of pulmonary hypertension was directly related to the activity of HO-1, rather than its expression.

**Conclusion:**

This study demonstrated that simvastatin treatment ameliorates established pulmonary hypertension primarily through an HO-1-dependent pathway.

## Background

Pulmonary hypertension (PH) is a rare but life-threatening disease characterized by significant increases in pulmonary arterial pressure (PAP) and right ventricular hypertrophy (RVH).[1] It is a disease of the small pulmonary arteries that results in a progressive increase in pulmonary vascular resistance and, ultimately, right ventricular failure and death.

Simvastatin, a 3-hydroxy-3-methylglutaryl coenzyme A (HMG-CoA) reductase inhibitor, is a statin, agents with well-known lipid-lowering effects that substantially decrease cardiovascular morbidity and mortality in patients with and without coronary artery diseases.[2,3] However, a growing body of evidence has revealed that the therapeutic benefits of statins cannot be explained solely by their inhibitory action on cholesterol synthesis.[4,5] These so-called pleiotropic, cholesterol-independent effects are believed to include anti-proliferative, anti-inflammatory, and antioxidant actions. Today, a number of experimental studies suggest the potential of statins as a novel therapy for PH, based on these pleiotropic effects.[6] Several experimental studies have investigated the possible mechanisms of simvastatin benefits in models of PH induced by monocrotaline (MCT) or by chronic hypoxia (CH). [7-9] However, the underlying mechanism of these protective effects of simvastatin in PH remains to be investigated.

Heme oxygenase (HO) is the rate-limiting enzyme in the oxidation of heme to biliverdin and carbon monoxide (CO). There are three isoenzymes of HO: HO-1, an inducible isoform, and HO-2 and HO-3, which are constitutively synthesized. Induction of the important cytoprotective molecule HO-1 has been shown to have vasodilatory, anti-inflammatory, and pro-apoptotic effects mediated through CO and bilirubin.[10,11] HO-1 expression is upregulated in several pulmonary diseases and can be induced by simvastatin treatment.[12] Previous studies have shown that raising endogenous HO-1 levels prevented CH-induced PH (CH-PH), and that HO-1 transgenic mice were protected from the development of PH and vessel wall hypertrophy induced by CH.[13,14] Moreover, HO-1 is a target site of statins in endothelial cells leading to HO-1 promoter activation, transcript and protein accumulation.[15] Simvastatin activates HO-1 expression through p38 and the phosphoinositide 3-kinase-Akt pathways which mediate its anti-inflammatory and anti-proliferative effects in vitro and in vivo.[16] Previously, our research group has also demonstrated that simvastatin could protect the liver from ischemia-reperfusion injury by HO-1 induction.[17] Therefore, we hypothesized here that simvastatin therapy could ameliorate PH severity in MCT-induced PH (MCT-PH) and CH-PH in the rat, and that HO-1 dependent pathways would be important mechanisms of the protective effects of simvastatin.

## Methods

### Animal Treatments and Groups

Female Wistar rats weighing between 200 and 250 g were used in our experiments. All animal care and experiments were performed in accordance with the Guide for the Care and Use of Animals (National Academy Press, Washington, DC, 1996). All protocols used in this study were approved by the Laboratory Animal Care Committee of National Taiwan University College of Medicine.

### Pulmonary Hypertension Animal Models

Rats in the MCT-PH groups were subcutaneously injected with monocrotaline (MCT, 60 mg/kg of body weight (kgw); Sigma-Aldrich, St. Louis, MO) on day 1. For 24 days, rats in the CH-PH groups were placed in an altitude chamber (hypobaric chamber) from 5 p.m. to 8 a.m. each day (intermittent exposure) and breathed air at normal (sea level) air pressure the rest of the time.[18] A simulated altitude of 5,500 m (380 torr) was selected because it represents the maximal altitude to which most rats can successfully adapt. Food and water were freely available at all times, and the animals were maintained at a constant temperature on a normal light cycle.

### Drug Treatment

**Figure 1:**
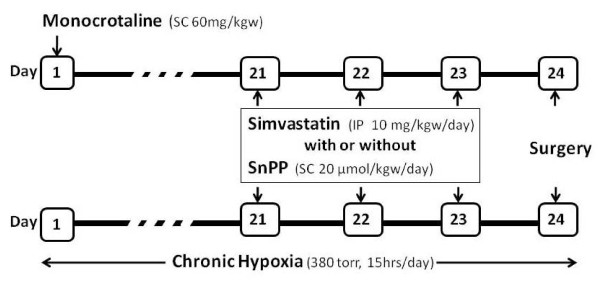
**Schematic outline of the experimental protocol**. Monocrotaline-induced pulmonary hypertension (MCT-PH) was established by subcutaneous administration on day 1 (60 mg/kgw), and chronic hypoxia-induced pulmonary hypertension (CH-PH) was established by treatment in a hypobaric chamber (380 Torr, intermittent exposure) for 24 days. Simvastatin was given by intraperitoneal injection (10 mg/kgw/day) on day 21~23, and SnPP (20 μmol/kgw/day) was injected alone or with simvastatin simultaneously on days 21~23. All animals underwent experimental surgery on day 24.

Simvastatin (10 mg/kgw; Calbiochem, San Diego, CA) was given daily by intraperitoneal (i.p.) injection for three days (days 21~23). Simvastatin was prepared as a solution in ethanol and then activated by alkaline hydrolysis. The final concentration was 5 mg/ml. Rats in the normal control group received only the same volume of solvent vehicle without simvastatin. Tin-protoporphyrin IX (SnPP, 20 μmol/kgw; Porphyrin Products, Inc., Logan, UT) was given by subcutaneous injection (s.c) on day 21~23 to inhibit HO activity. (Figure 1)

### Animal Groups

The experimental animals were divided into the following 10 groups (each group *n *≥ 4, see Table 1): normal control (N), normal + simvastatin treatment (NS), monocrotaline treatment (M), monocrotaline + simvastatin treatment (MS), monocrotaline + simvastatin + SnPP treatment (MSP), monocrotaline + SnPP treatment (MP), chronic hypoxia (H), chronic hypoxia + simvastatin treatment (HS), chronic hypoxia + simvastatin + SnPP treatment (HSP), chronic hypoxia + SnPP treatment (HP).

**Table 1:** Pulmonary hypertension rat models and pharmacologic treatment in the ten experimental groups

**Group (Abbreviation)**	**Treatment**
Normal control (N)	Sham
Normal+Simvastatin (NS)	Simvastatin on days 21~23

MCT (M)	MCT on day 1
MCT+Simvastatin (MS)	MCT on day 1; simvastatin on days 21~23
MCT+Simvastatin+SnPP (MSP)	MCT on day 1; co-treated with simvastatin and SnPP on days 21~23
MCT+SnPP (MP)	MCT on day 1; SnPP on days 21~23

Chronic Hypoxia (H)	Placed in a hypobaric chamber for 24 days
Chronic Hypoxia+Simvastatin (HS)	Chronic hypoxia for 24 days, simvastatin on days 21~23
Chronic Hypoxia+Simvastatin+SnPP (HSP)	Chronic hypoxia for 24 days, co-treated with simvastatin and SnPP on days 21~23
Chronic Hypoxia+SnPP (HP)	Chronic hypoxia, SnPP on days 21~23

### Measurement of Pulmonary Arterial Pressure

On day 24, rats were anesthetized with pentobarbital (35 mg/kgw, i.p. injection). Tracheal cannulation was performed after tracheostomy. The chest was opened via a midline incision, and the animal was ventilated by a ventilator with room air (70 strokes/min, tidal volume 10 ml/kg), with about 2–3 cm H_2_O positive end expiratory pressure (PEEP). The right ventricle outflow tract was directly punctured by a polyethylene tubing PE-10 tube. Subsequently, a silastic catheter was inserted via the marks created by the puncture wound to monitor pulmonary artery blood pressure. Polyethylene cannulas were placed in the left femoral vein for infusion of saline at 1.2 ml per hour, the left femoral artery for blood sampling, and the left carotid artery for measurement of mean arterial pressure.

### Western Blot Analysis

For detection of HO-1 immunoreactive proteins, lung tissues from each group were homogenized in T-PER tissue protein extraction buffer (Pierce, Rockford, IL) containing protease inhibitors. The lysates were separated by 12% sodium dodecyl sulfate-polyacrylamide gel electrophoresis and then blotted and stained with HO-1 antibody (OSA-111; StressGen, Victoria, B.C., Canada) following procedures previously described.[17] Protein signal quantification was performed by computer-assisted densitometry (Gel Pro 3.1; Media Cybernetics, Bethesda, MD).

### HO Activity Assay

HO activity in lung cells of each group was measured by the generation of bilirubin. [19-21] Briefly, microsomes from lung tissue were added to a reaction mixture containing NADPH, rat liver cytosol as a source of biliverdin reductase, and the substrate hemin. The reaction was conducted at 37°C in the dark for 1 hr and terminated by the addition of 0.5 mL of chloroform, and the extracted bilirubin was calculated by the difference in absorbance between 460 and 530 nm with an extinction coefficient of 40 mM^-1^cm^-1^. The HO activity was expressed as picomoles per hour per milligram of protein, and then normalized to the control group.

### Immunohistochemistry

Four-micrometer lung sections were deparaffinized in xylene and then rehydrated in graded alcohol solutions. To determine the distribution of HO-1 protein, the lung sections were incubated with the OSA-111 HO-1 antibody (StressGen). The detailed procedure was as previously described.[17]

### Statistical Methods

Data are expressed as means ± S.E. Statistical analysis was performed using Sigma Stat software (Jandel Scientific, San Raphael, CA) by unpaired Student's *t*-test for comparisons between 2 groups, and by one-way analysis of variance (ANOVA) for comparisons between >2 groups. A *p *value < 0.05 was regarded as significant.

## Results

### Two Rat Models of Pulmonary Hypertension

**Figure 2:**
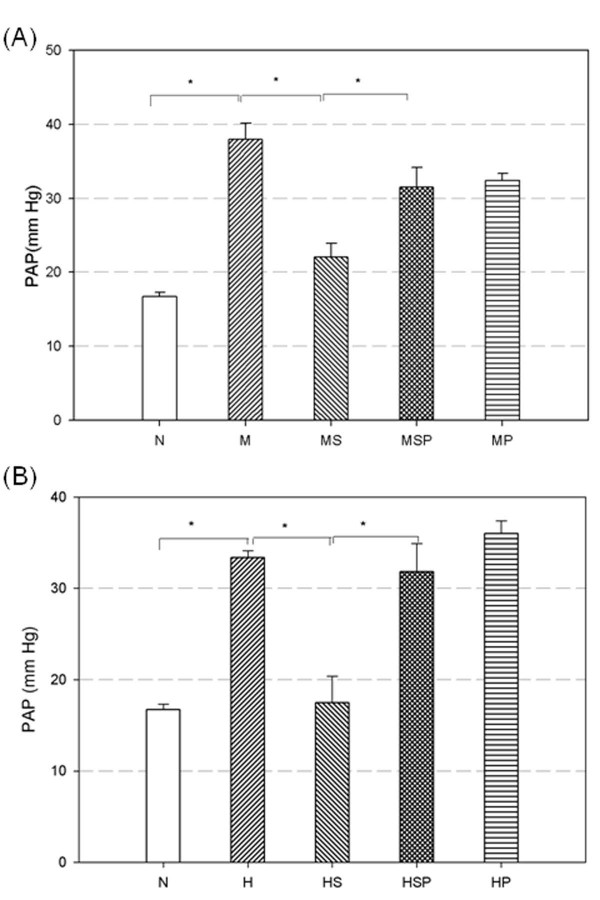
**Effects of simvastatin and SnPP on pulmonary arterial pressure in MCT-PH (A) and CH-PH (B) rats**. Rats that received simvastatin (MS and HS groups) had significantly decreased pulmonary arterial pressure compared with non-simvastatin treated PH rats (M and H groups). Co-treatment with simvastatin and SnPP (MSP and HSP groups) failed to relieve the high pulmonary arterial pressure. Rats that received SnPP alone (MP and HP groups) maintained high pulmonary arterial pressure. Horizontal bars indicate groups that differed significantly from each other, * *p *< 0.05. (N = normal control, M = monocrotaline treatment, MS = monocrotaline + simvastatin treatment, MSP = monocrotaline + simvastatin + SnPP treatment, MP = monocrotaline + SnPP treatment, H = chronic hypoxia, HS = chronic hypoxia + simvastatin treatment, HSP = chronic hypoxia + simvastatin + SnPP treatment, HP = chronic hypoxia + SnPP treatment).

MCT administration (M group) significantly increased the PAP (38.0 ± 2.2 mm Hg) on day 24 compared with the normal control group (16.7 ± 0.6 mm Hg for N group, *p *< 0.05, Figure 2A). Twenty-four days of CH (H group) also induced a significant increase in PAP (33.3 ± 0.8 mm Hg, *p *< 0.05 versus N group, Figure 2B). However, there was no significant difference in PAP between the MCT and CH groups. In addition, no significant change in systemic blood pressure was found between the normal control (N group) and the MCT or CH groups (data not shown).

### Simvastatin Ameliorated Established Pulmonary Hypertension

In preliminary experiments, we treated PH rats with simvastatin for 1, 2, and 3 days and examined the severity of pulmonary hypertension. Established pulmonary hypertension induced by both MCT and CH were significantly decreased by 3 days of simvastatin treatment but not by treatment for 1 or 2 days (data not shown). After 3 days of treatment (days 21 ~23) with simvastatin (10 mg/kgw, i.p.) in MCT-PH rats (MS group), the PAP measured on day 24 was significantly decreased to 22.1 ± 1.9 mm Hg (*p *< 0.05 vs M group, Figure 2A). In CH-PH rats, 3-day administration of simvastatin (HS group) significantly decreased the PAP (17.5 ± 2.9 mm Hg) on day 24 compared with CH-PH rats (H group, *p *< 0.05, Figure 2B). However, there was no change in systemic blood pressure before and after simvastatin treatment in these groups (data not shown).

### Co-treatment with SnPP and Simvastatin Maintained High Pulmonary Arterial Pressure

To investigate the role of HO-1 in the pulmonary arterial pressure lowering effect of simvastatin treatment, the heme oxygenase (HO) activity competitive inhibitor, SnPP (20 μmol/kgw, s.c, on day 21~23), was administered alone or simultaneously with simvastatin to MCT-PH and CH-PH rats. MCT-PH rats given SnPP alone (MP group) maintained a high PAP (32.4 ± 0.9 mm Hg, Figure 2A). Co-administration of simvastatin and SnPP in MCT-PH rats (MSP group) significantly abolished the effects of simvastatin (31.5 ± 2.7 mm Hg, *p *< 0.05 versus the MS group, Figure 2A). In the CH-PH rats group, the PAP after SnPP treatment (HP group) was 36.0 ± 1.4 mm Hg (Figure 2B). Although simvastatin treatment could decrease PAP in rats with chronic hypoxia (HS group, Figure 2B), co-treatment with SnPP (HSP group) abolished the simvastatin effect (31.8 ± 3.1 mm Hg for HSP group, *p *< 0.05 versus the HS group, Figure 2B). Co-treatment with SnPP did not change the systemic blood pressure in these groups (data not shown). The PAP lowering effects of simvastatin in MCT-PH and CH-PH rats were suppressed by co-treatment with the HO inhibitor, SnPP.

### Effect of Simvastatin on Right Ventricular Hypertrophy

**Figure 3:**
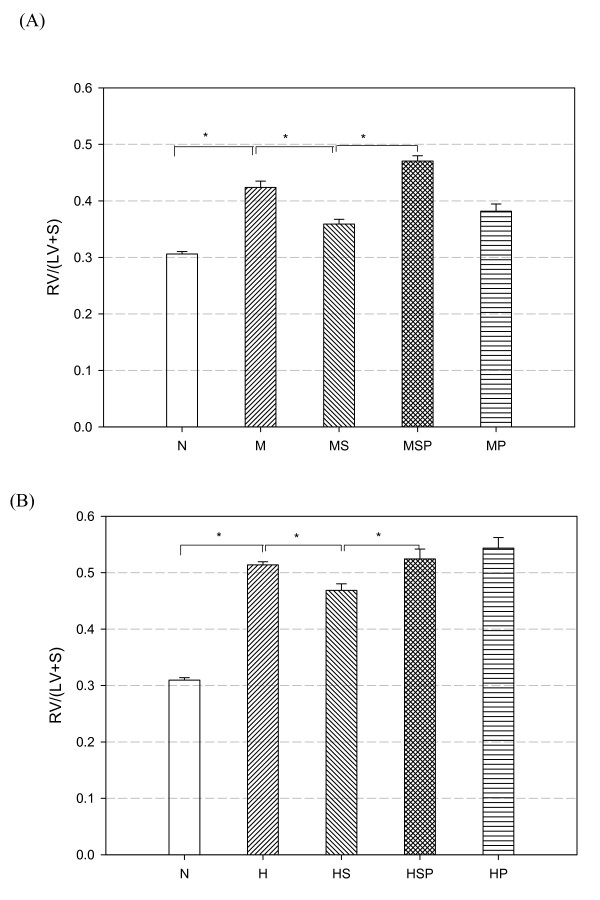
**The effects of simvastatin and SnPP on right ventricular hypertrophy in MCT-PH (A) and CH-PH (B) rats**. Simvastatin treatment (MS and HS groups) prevented progression and improved established right ventricular hypertrophy compared with the non-simvastatin treated PH rats (M and H groups). Rats that received SnPP alone (MP and HP groups) or received both simvastatin and SnPP (MSP and HSP groups) developed severe right ventricular hypertrophy. The index of right ventricular hypertrophy is given as the ratio of right ventricle to (left ventricle plus septum) weight [RV/(LV+S)]. Horizontal bars indicate groups that differed significantly from each other, * *p *< 0.05. (N = normal control, M = monocrotaline treatment, MS = monocrotaline + simvastatin treatment, MSP = monocrotaline + simvastatin + SnPP treatment, MP = monocrotaline + SnPP treatment, H = chronic hypoxia, HS = chronic hypoxia + simvastatin treatment, HSP = chronic hypoxia + simvastatin + SnPP treatment, HP = chronic hypoxia + SnPP treatment).

Right ventricular hypertrophy (RVH) is a hallmark of PH. The ratio of right ventricular to left ventricular plus septum weight (RV/LV+S) is a commonly used indicator of the severity of PH. In this study, the RV/LV+S ratios in MCT-PH (0.42 ± 0.01) and CH-PH (0.51 ± 0.005) rats were both significantly higher than in normal control (0.30 ± 0.004; both M:N and H:N *p *< 0.05, Figure 3). After three days of simvastatin treatment, the RV/LV+S ratio fell significantly to 0.36 ± 0.009 (MS group; *p *< 0.05 versus the M group, Figure 3A) and 0.47 ± 0.011 (HS group; *p *< 0.05 versus the H group, Figure 3B). These data showed that simvastatin treatment could significantly decrease the RV/LV+S ratio in PH rats, resulting from a reduction in right ventricular work.

### SnPP Suppressed the Ameliorative Effect of Simvastatin on RVH

Rats co-treated with simvastatin and SnPP in both MCT-PH and CH-PH protocols maintained high RV/LV+S ratios. The RV/LV+S ratio was 0.47 ± 0.009 in the MSP group (*p *< 0.05 versus MS group, Figure 3A) and 0.52 ± 0.017 in the HSP group (*p *< 0.05 versus the HS group, Figure 3B).

### Effect of Simvastatin on HO-1 Protein Expression in Lung

**Figure 4:**
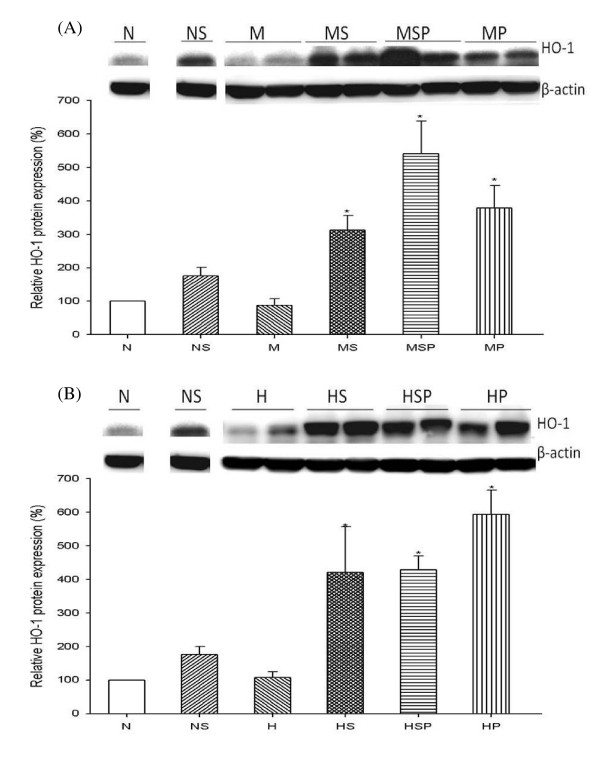
**Western blot analysis of HO-1 protein expression in MCT-PH (A) and CH-PH (B) rat lung**. Densitometric quantification revealed a significant increase in expression of HO-1 protein in the lungs of rats that received simvastatin and SnPP treatment. Densitometric units were normalized to β-actin and then divided by N group results. (N = normal control, NS = normal + simvastatin treatment, M = monocrotaline treatment, MS = monocrotaline + simvastatin treatment, MSP = monocrotaline + simvastatin + SnPP treatment, MP = monocrotaline + SnPP treatment, H = chronic hypoxia, HS = chronic hypoxia + simvastatin treatment, HSP = chronic hypoxia + simvastatin + SnPP treatment, HP = chronic hypoxia + SnPP treatment).

**Figure 5:**
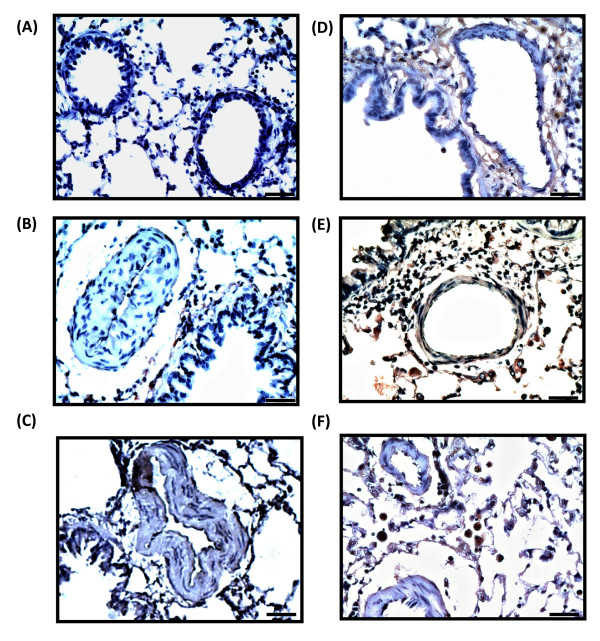
**Immunohistochemical staining of the distribution of HO-1 in rat lung**. Simvastatin treatment led to marked HO-1 immunoreactivity around pulmonary vascular sites. (A) normal control, (B) MCT-PH rats, (C) CH-PH rats, (D) normal rats treated with simvastatin, (E) simvastatin treated, MCT-PH rats, (F) simvastatin treated, CH-PH rats. Magnification: 400×. Bars represent 20 μm.

To investigate the relationship between HO-1 expression and the simvastatin attenuation of established PH, we examined the level and distribution of HO-1 protein in rat lung. HO-1 protein expression increased after simvastatin treatment in the normal control group (NS group, Figure 4). In MCT-PH and CH-PH rats, simvastatin administration led to significantly higher levels of HO-1 protein expression, of 310 ± 40% (MS group, *p *< 0.05 versus M group, Figure 4A) and 420 ± 130% (HS group, *p *< 0.05 versus H group, Figure 4B). Interestingly, treatment of PH rats with SnPP alone (MP and HP groups) or co-administration of SnPP and simvastatin (MSP and HSP groups) also significantly increased HO-1 protein expression (*p *< 0.05 for MP group vs. M group, MSP group vs. M group, HP group vs. H group, and HSP group vs. H group). Furthermore, immunohistochemical staining revealed that the distribution of HO-1 protein expression increased around the pulmonary vascular sites after simvastatin treatment (Figure 5).

### Effect of Simvastatin on Heme Oxygenase Activity in Rat Lung

**Figure 6:**
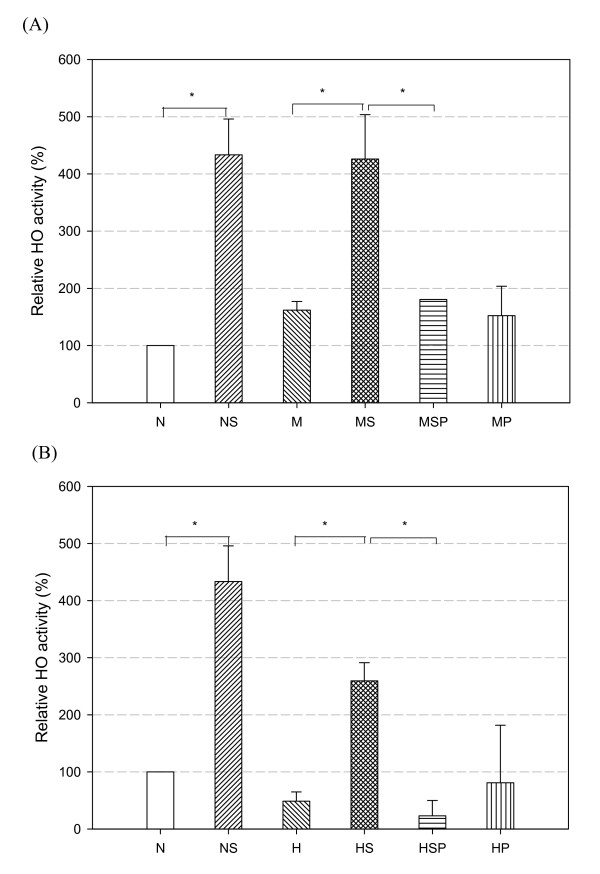
**Changes in HO activity in MCT-PH (A) and CH-PH (B) rat lungs**. Simvastatin treatment (MS and HS groups) significantly increased HO activity compared with non-simvastatin treatment in PH rats (M and H groups). Co-administration of simvastatin and SnPP (MSP and HSP groups) led to significantly less HO activity than in simvastatin-treated rats (MS and HS groups). The bilirubin concentration in each group was normalized to that of normal controls (Group N, value = 1.0). Horizontal bars indicate groups that differed significantly from each other, * *p *< 0.05.

The effect of simvastatin treatment on heme oxygenase (HO) activity in rat lung was measured, based on bilirubin generation. The bilirubin concentration in each group was normalized to that of the normal control group. In MCT-PH and CH-PH rats, simvastatin treatment caused a significant elevation in HO activity from 130 ± 50% of control (M group) to 280 ± 50% of control (MS group, *p *< 0.05 vs. M group, Figure 6A) and from 150 ± 40% of control (H group) to 280 ± 40% of control (HS group, *p *< 0.05 vs. H group, Figure 6B). After co-treatment with SnPP, the relative increase of HO activity compared to control decreased to 130 ± 20% (MSP group, *p *< 0.05 vs. the MS group, Figure 6A) and 110 ± 10% (HSP group, *p *< 0.05 versus the HS group, Figure 6B). In MCT-PH rats, the HO activities in the SnPP-treated rats (MSP and MP groups) were not significantly different (*p *= 0.583 in MSP group versus N group, *p *= 0.712 in MP group versus N group, Figure 6A). These results show that simvastatin significantly increased HO activity in PH rat lungs, and this effect was blocked by co-administration of SnPP.

## Discussion

In this study, we demonstrated that simvastatin treatment decreased the pulmonary hypertension induced by MCT or chronic hypoxia in rats via a mechanism which was dependent on HO-1 activity. The main findings of this study are as follows: (a) simvastatin could ameliorate pulmonary hypertension in MCT-PH or CH-PH rats; (b) both levels and activity of HO-1 were increased in rat lung after simvastatin treatment; (c) the therapeutic effect of simvastatin was abolished by inhibiting HO-1 enzyme activity with SnPP, suggesting that the therapeutic effect of simvastatin is mediated by HO-1. This study demonstrated that HO-1 activity plays a key role in the attenuation of established pulmonary hypertension by simvastatin treatment in PH rats. To our knowledge, this is the first study to show that the decrease in severity of pulmonary hypertension in rat lungs after simvastatin treatment depends on the HO-1 enzyme activity rather than the level of HO-1 protein expression.

Although numerous researchers prefer to use male animals for PH experiments to avoid any potential influence of female hormones on the study results, we chose female rats in this study for the potential application of simvastatin in clinical use. Current literature describes nearly no male-dominant tendencies in most clinical PH diseases in humans.[22] In contrast, the incidence and prevalence of idiopathic pulmonary arterial hypertension (PAH) and secondary PAH related to connective tissue diseases in human is higher in females than in males. [23-25] We feel, therefore, that experimental results from female rats are both clinically valid and necessary.

Previous reports have described that the beneficial effects of simvastatin in preventing or reversing established PH were observed over weeks of oral simvastatin administration.[8,26] In our study, significant attenuation of established pulmonary hypertension was seen with treatment times as short as three days, via intraperitoneal injection. Although short duration oral simvastatin treatment may also have the same effects as intraperitoneal injection, at this point in time the short-term effects of oral simvastatin on PH animals have not been examined. One reasonable explanation for the prolonged treatment duration required following oral simvastatin administration was that the absorption and distribution of simvastatin were slower than following intraperitoneal administration. Although this intraperitoneal administration differs from the more normal oral administration used in clinical settings, our results still have valid clinical implications.

We examined the effects of simvastatin in two well-established PH animal models, MCT- and CH-PH rats. MCT is a pyrrolizidine alkaloid which is activated in the liver and then transfers to the lungs causing pulmonary injury and trauma.[27] In previous studies, pulmonary arterial endothelial injury followed by an inflammatory response triggered severe, progressive pulmonary hypertension, as indicated by a dramatic increase in PAP and RVH.[28,29] On the other hand, CH exposure leads to structural changes in the pulmonary arteries, including smooth muscle proliferation and hypertrophy, and produces well-known adaptive PH changes in rat lungs. [30-33] Simvastatin therapy can provide anti-inflammatory, anti-proliferative, and pro-apoptotic effects and has been well demonstrated to prevent or attenuate established PH in several PH rat models. Simvastatin may restore eNOS expression in endothelial cells, and can also attenuate PH and inhibit pulmonary vascular remodelling in CH rats.[34,35] In addition, simvastatin was able to rescue rats from fatal PH by inducing apoptosis of vascular neointimal smooth muscle cells in a pneumonectomy-plus-MCT treatment rat model.[26] A recent study indicated that simvastatin may ameliorate CH-PH by the inhibition of ROCK expression and activity.[8] However, the exact mechanism by which simvastatin mediates protection against PH has not yet been fully elucidated.

In the human body, heme oxygenase-1 (HO-1) degrades heme to generate carbon monoxide (CO), iron, and biliverdin, which is subsequently converted to bilirubin. HO-1 is considered to be a cytoprotective enzyme because each of these products of heme breakdown plays its own protective role. Recent studies have found that inducible HO-1 regulates vascular tone and cell proliferation through the production of endogenous CO by the degradation of heme.[36] CO activates guanylate cyclase, raising cellular cGMP level and resulting in vascular smooth muscle relaxation.[37] Increasing evidence also suggests a regulatory interaction in vascular smooth muscle cells between CO and NO via HO-1, with HO-1 being regulated by NO during hypoxia.[38,39] Lee et al. demonstrated that the anti-inflammatory and anti-proliferative effects of simvastatin were largely through the simvastatin-induced HO-1 and the potential mechanism activating HO-1 expression may be through p38 and phosphoinositide 3-kinase-Akt pathways.[16] Taken together, we suggest that HO-1 may be crucial in the simvastatin effects of attenuating PH.

There is other accumulating evidence that supports the idea that HO-1 plays an important role in PH.[40] Lack of HO-1 expression in HO-1-knockout mice was associated with organized mural thrombi and subsequent PH; HO-1 transgenic mice were protected from the development of vessel wall hypertrophy induced by CH.[14,41] HO-1 may play defensive roles against MCT-induced pulmonary inflammation and cardiac hypertrophy in mice.[42] Enhancement of endogenous HO-1 by NiCl_2 _or hemin could prevent CH-PH.[13] Furthermore, previous studies have shown that simvastatin induced HO-1 protein expression in vascular smooth muscle cells [16] and endothelial cells.[15] Microarray analysis revealed that HO-1 was one of the genes with significantly increased expression after simvastatin treatment in CH-PH rats.[43] Zhou et al. showed that augmentation of endogenous HO-1 by rapamycin treatment had antiproliferative and vascular protective effects in MCT-PH.[44] These findings indicate that the enhancement of HO-1 provides a therapeutic effect in experimental pulmonary hypertension. Therefore, we proposed that the HO-1 pathway is involved in simvastatin amelioration of pulmonary hypertension. Our results confirm that induction of HO-1 by simvastatin was responsible for attenuating effects on PH and the severity of RVH in MCT- and CH-induced PH rats.

In order to elucidate the important role of HO-1 in mediating the protective effects of simvastatin, we co-administered simvastatin with tin-protoporphyrin (SnPP), a competitive antagonist of HO, in PH rats.[45] SnPP binds to the catalytic sites of heme oxygenase to block the activity of HO proteins, thus permitting its use as a pharmacological agent for suppressing heme catabolism in animal models.[46] Administration of simvastatin with SnPP significantly decreased HO activity and thus successfully abolished the therapeutic effects of simvastatin in PH rats. Because HO-2 expression is constitutive and only HO-1 expression is inducible, it appears that simvastatin-induced HO-1 protein expression is likely the main contributor to the change of HO activity. Zhou et al. demonstrated a similar effect of co-treatment with SnPP on rapamycin-induced HO-1-mediated antiproliferative effects in vascular disease.[44] Interestingly, although SnPP administration in PH rats in the present study had no beneficial effect on pulmonary hypertension, the presence of SnPP still significantly increased HO-1 protein expression compared with that of untreated PH rats. This phenomenon may be resulted from the dual mechanism whereby SnPP regulates heme oxygenase – potently inhibiting the enzyme at the catalytic site by acting as a competitive substrate for heme while enhancing the synthesis of new enzyme protein[47]. Sardana et al. also demonstrated that HO-1 levels increased strongly in rat liver after SnPP application while HO-1 activity was significantly inhibited.[47] Based on these findings, we hypothesize that HO-1 activity rather than HO-1 protein over-expression is the key factor underlying the beneficial effects of simvastatin treatment in MCT- and CH-PH.

There were several limitations of this study. First, the mechanism responsible for human PH remains to be elucidated and our two animal models only partially mimic the pathologic changes which occur in these conditions. Therefore, it remains unclear whether the clinical benefits of simvastatin in PH will be consistent with our results in these animal models. Second, while we used a simvastatin dose (10 mg/kgw) over a short duration (once daily treatment for 3 days) which did effectively reduce the PAP in PH rats, determination of the best dose and frequency of simvastatin treatment in clinical applications requires further investigation. Third, while we did not evaluate the effect of simvastatin on pulmonary vascular remodelling in PH rats because we did not anticipate any significant change to the pulmonary vasculature in this short duration of simvastatin treatment, this also remains untested.

## Conclusion

This study demonstrated that simvastatin could attenuate the severity of established PH in both MCT- and CH-PH rat models and that the mechanism underlying this beneficial effect is mediated by HO-1 activity rather than expression.

## Competing interests

The authors declare that they have no competing interests.

## Authors' contributions

HHH conceived the study, analysis, interpretation and drafted the manuscript. WJK and CFC participated in the design and coordination of the study, data acquisition and critical revision of the manuscript. JYH participated in the design, performed the analyses and helped to draft the manuscript. JSC, YCL, and IRL participated in the design and provided expert consultation. All authors read and approved the final manuscript.
